# Optimization of tea‐leaf saponins water extraction and relationships between their contents and tea (*Camellia sinensis*) tree varieties

**DOI:** 10.1002/fsn3.724

**Published:** 2018-07-30

**Authors:** Xiao‐Lan Yu, Yong He

**Affiliations:** ^1^ College of Biosystems Engineering and Food Science Zhejiang University Hangzhou China

**Keywords:** liquid–solid ratio, tea tree variety, tea‐leaf saponins, water extraction

## Abstract

Resulting from the year‐on‐year increase in tea plantations and the saturated consumption of tea leaves, the relative overcapacity in China's tea‐leaf production appears. Discovering the new utilization of tea leaves is helpful to alleviate this phenomenon. The feasibility of extracting saponins from aged tea leaves was investigated and confirmed; three major variables in water extraction were optimized by Box‐Behnken designs. The significant variable found in Box‐Behnken designs, liquid–solid ratio, was went through single‐variable experiments for a more accurate optimization. Seventy‐five ml/g, 1 hr, and 80°C were optimal values and tea‐leaf saponins yield of tea tree variety Longjing 43 reached 12.19% ± 0.0030% after optimizations, higher than the yield of tea‐seed saponins from *Camellia oleifera* seed meals using the same extraction method (water extraction based on optimizations). According to correlation analyses, tea tree's leaf type and germination stage affected tea‐leaf saponins contents positively, indicating tea trees with larger leaves and later germination stage would have a higher content of tea‐leaf saponins with a higher yield of tea‐leaf saponins under the same extraction method.

## 1 INTRODUCTION

Tea, made from tender shoots and leaves of tea (*Camellia sinensis*) trees, is one of the most consumed beverages all over the world, making tea (*C. sinensis*) become an important economic crop in China. The past few years have witnessed the continuous growth of tea plantations in China while the consumption capacity of tea tends to be steady, leading to the relative surplus of tea leaves, and meanwhile, due to the recognition and enthusiasm of Chinese market for the freshness and tenderness of tea leaves, Chinese tea farmers usually only pick fresh and tender shoots and leaves of tea trees in spring, with aged leaves disused and grown without management, which aggravated the relative surplus phenomenon. Therefore, to make the best use of tea leaves, specially aged leaves, is a principal measure to solve the phenomenon.

Besides tea polyphenols, caffeine and catechins, tea saponin, another secondary metabolite of tea trees, gradually becomes the attention of researchers because of its effects of hemolysis, antibacterial, anti‐inflammatory, anti‐oxidation along with inhibition of alcohol absorption (Lv, Qu, Sun, & Li, 2005; Sun, Cai, Liang, & Yang, 2017; Wen, Lu, Jiang, Yan, & Fang, 2011; Yan, Wei, Xu, Li, & Guo, 2014; Zhao, Xue, Yang, & Wei, 2010). Moreover, as a natural nonionic surfactant, tea saponin is not only used in the manufacture of cleaning products (Feng, Chen, Liu, & Liu, 2015; Li, Wu, Yang, & Yi, 2016; Mao, Qi, Sun, Zeng, & Yan, 2016; Wu, Huang, Huang, Wang, & Song, 2017), but also in soil remediation (Cay, 2016; Liu, Cao, Wang, Zhang, & Hu, 2017; Wang et al., 2016; Ye et al., 2015, 2017). Contained in stems, leaves, flowers, and seeds of tea (*C. sinensis*) (Lu, Ning, Fang, Jiang, & Wei, 2012; Ribeiro, Coelho, Rebelo, & Marrucho, 2013; Xiong et al., 2016) as well as other plants of the genus *Camellia*, such as *Camellia oleifera* and *Camellia chekiang‐oleosa* Hu, tea saponin is classified into tea‐leaf saponins (foliatheasaponins) (Morikawa, Matsuda, Li, Li, & Yoshikawa, 2007; Morikawa, Nakamura, et al., 2007; Sagesaka, Uemura, Watanabe, Sakata, & Uzawa, 1994) and tea‐seed saponins (theasaponins) (Morikawa, Matsuda, et al., 2007; Yoshikawa et al., 2007; Yoshikawa et al., 2005) due to its distribution, also, researches have proven that there are some differences between the composition of tea‐leaf saponins and tea‐seed saponins (Morikawa, Matsuda, et al., 2007; Morikawa, Nakamura, et al., 2007; Sagesaka et al., 1994; Wan, 2011; Yoshikawa et al., 2007; Yoshikawa et al., 2005). At present, the clear majority of saponins are extracted from *C. oleifera* seed meals (He et al., 2014; Hu, Nie, Huang, Li, & Xie, 2012; Yang et al., 2015), the by‐product from oil extractions. There is less research on the extraction of saponins from tea leaves or factors affecting the content of tea‐leaf saponins. However, it is feasible and necessary to investigate the extraction yield of saponins from tea leaves and factors influencing tea‐leaf saponins contents, which benefits to deepen the understanding of tea leaves and contributes to the comprehensive utilization of excessive tea leaves.

Among extraction methods for tea‐seed saponins, for example, water extraction (Chen, Xiao, Huo, Long, & Liu, 2017; Li, Wu, et al., 2016; Li, Yu, et al., 2016), organic solvent extraction (Hu et al., 2012; Liu, Quan, Huang, Chen, & Zhang, 2013; Yi et al., 2016; Yu, Chen, Wu, & Ren, 2013), mixed solvent extraction (Shi et al., 2011), microwave‐assisted extraction (Gong, Liang, Zhang, & Xiao, 2013; He et al., 2014; Peng, Zhou, & Guo, 2009), ultrasonic‐assisted extraction (He, Zhang, & Zhang, 2015; Qi & Zhang, 2014; Zhang, Hu, Li, & Deng, 2012), supercritical extraction (Xiaoling Lv & Li, 2005), aqueous enzymatic (Wang, 2013; Zhang & Wang, 2014; Zhou & Yang, 2016) and fermentation (Wang et al., 2014), water extraction is definitely the simplest, easiest, and greenest measure. The problem of low yield might exist in water extraction, whereas it could be solved by optimizing the variables in water extraction, such as liquid–solid ratio, extraction time, and extraction temperature, whose major effects on the yield of saponins were verified by studies (Gong et al., 2017; He et al., 2014; Hu, Nie, Gong, Li, & Xie, 2009; Hu et al., 2012; Li, Wu, et al., 2016; Li, Yu, et al., 2016; Qi & Zhang, 2014; Zhan & Xie, 2012; Zhang, Qian, Zhang, & Fan, 2003; Zhu, Lin, Chen, Xie, & Wang, 2011), not just in water extraction, but in organic solvent extraction and other extraction methods.

This research aimed at investigating the feasibility of extracting tea‐leaf saponins from tea (*C. sinensis*) trees with the assistance of water extraction and at the same time, optimizing three major variables (liquid–solid ratio, extraction time, and extraction temperature) in water extraction to acquire a higher yield of tea‐leaf saponins. Factors impacting the content of tea‐leaf saponins between different tea tree varieties were also analyzed and discussed.

## 2 MATERIALS AND METHODS

### 2.1 Materials and chemicals

Six varieties of tea trees, namely Longjing 43, Anji white tea, Zisun, Huangjinya, Jiukengzao, and Zhenong 117 with details given in Table 1, were chosen and their first leaves on the last year's twig (from top to down) were picked as aged leaves from a pollution‐free tea plantation [grant number: WNCR‐ZJ16‐12141] in Changxing County, Zhejiang Province, China on December 22, 2017.

**Table 1:** Information on six varieties of tea trees

Variety	Leaf type	Germination stage	Adaptability
Longjing 43	Medium (1)	Extremely early (1)	Green tea (1)
Anji white tea	Medium (1)	Medium (3)	Green tea (1)
Zisun	Medium (1)	Medium (3)	Green & black tea (2)
Huangjinya	Medium (1)	Medium (3)	Green tea (1)
Jiukengzao	Large (2)	Early (2)	Green tea (1)
Zhenong 117	Medium (1)	Early (2)	Green tea (1)

Note

Information acquired from *Tea Tree Cultivation* (Luo 2013).

All leaves were wiped to remove dust and then dried in an oven at 60 °C until they were in constant weights and sieved through a No. 60 mesh variety‐by‐variety.

Chemicals and reagents of analytical grade bought from Sinopharm Chemical Reagent Co., Ltd, Shanghai, China were used in this research. Deionized water (≥18.2 MΩ) was utilized for the preparation of aqueous solutions.

### 2.2 Methods

The well‐established vanillin‐sulfuric acid method (Makkar & Becker, 2007) was employed to establish the standard curve and to determine the content of saponins in extraction solutions with the aid of a UV–visible spectrophotometer (Cary 60 UV‐Vis; Agilent Technologies, USA).

Three‐level Box‐Behnken designs were designed to optimize three major variables in tea‐leaf saponins water extraction, which were liquid–solid ratio (ml/g), extraction time (hr), and extraction temperature (°C) with ranges designed consulting existing literatures (Qi & Zhang, 2014; Zhang et al., 2003). The variety, Longjing 43, was selected for optimization experiments due to its popularity and universality in Zhejiang Province, and 0.5 g was set as the amount for each measurement. Because the real content of tea‐leaf saponins could not be determined for the moment, making the extraction rate, which used the real content of tea‐leaf saponins as the denominator, not applicable for comparing the performances of optimizations. Thus, the yield (%), which used the mass of tea‐leaf powder as the denominator, was applied as the dependent variable in Box‐Behnken designs. The yield (%) of tea‐leaf saponins was calculated as equation (1):

(1) $$\text{yield}\;\left(\%\right)=\frac{c_{\mathrm{saponin}}\times V_{\mathrm{extraction}\;\mathrm{solution}}}{m_{\mathrm{tea}\;\mathrm{powder}}}\times 100\%,$$

where *c*_saponin_ was the concentration of each extraction solution (g/ml) and calculated from the standard curve and the dilution factor, *V*_extraction solution_ was the volume of each extraction solution (ml) obtained by the measuring cylinder after a 0.45‐μm microporous filtering film and *m*_tea powder_ was the mass of tea‐leaf powder utilized for each extraction (g) and determined by an electronic balance accurately (BSA224S; Sartorius scientific instruments (Beijing) Co., Ltd.). The significant level was 0.05, and data acquired from Box‐Behnken designs were analyzed by Design‐Expert 11 (Stat‐Ease, Inc., USA).

For significant variable without significant interactions in Box‐Behnken designs, single‐variable experiments were employed as an auxiliary method to define the effect of this variable more accurately on tea‐leaf saponins yields.

Tea‐leaf saponins yields of other five tea tree varieties were obtained by the optimized water extraction method. The extraction and measurement were performed three times for each variety.

### 2.3 Statistical analysis

Independent sample *t* test of tea‐leaf saponins yields from six varieties of tea trees as well as the correlation between yields and leaf type, germination stage along with adaptability was carried out and presented by SAS University Edition (SAS Institute Inc., USA).

## 3 RESULTS AND DISCUSSION

### 3.1 Box‐Behnken designs for analysis and optimization

**Figure 1:**
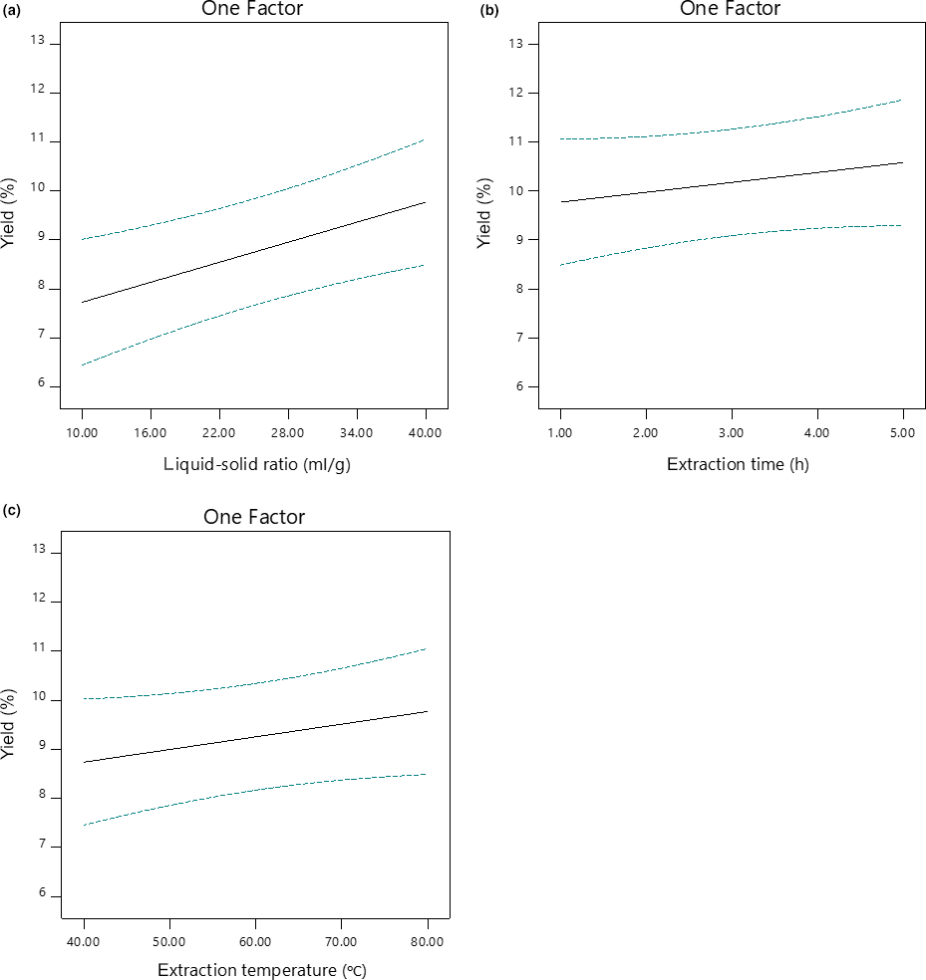
Effects of optimized variables on tea‐leaf saponins yields by Box‐Behnken designs

Demonstrated in Tables 2 and 3, it was clear that the linear model, the variable liquid–solid ratio and *Lack of Fit* all reached significant level, especially the variable liquid–solid ratio and *Lack of Fit*, arriving at the extremely significant level with *p*‐value <0.01 and no interactions were found significant. The effect of liquid–solid ratio, extraction time, and extraction temperature on the yield of tea‐leaf saponins was similar: the yield of tea‐leaf saponins increased as liquid–solid ratio, extraction time, and extraction temperature increased in the range of this study, illustrated in Figure 1. Taking the significance of these three variables into account, the reduced linear model using backward regression (α < 0.05) was carried out to eliminate redundant variables, and as Table 4 shows, results were as expected: the *Lack of Fit* of the reduced linear model was improved. Besides, liquid–solid ratio's extreme significance indicated the necessity of additional single‐variable experiments to clarify its impacts on the yield of tea‐leaf saponins in a larger range.

**Table 2:** Box‐Behnken designs for tea‐leaf saponins water extraction

Run	Liquid–solid ratio (ml/g)	Extraction time (hr)	Extraction temperature (°C)	Yield (%)
1	25	5	80	9.48
2	10	3	40	7.23
3	25	3	60	8.79
4	25	1	80	9.17
5	40	3	80	8.88
6	10	1	60	7.66
7	10	3	80	7.72
8	40	5	60	12.18
9	25	3	60	8.87
10	25	3	60	8.74
11	10	5	60	7.64
12	25	5	40	7.95
13	25	1	40	7.88
14	40	3	40	8.06
15	40	1	60	9.31

**Table 3:** Analysis of Variance (ANOVA) for the linear model selected from Box‐Behnken designs

Source	Sum of squares	*df*	Mean square	*F* value	*p*‐Value
Model	11.80	3	3.93	5.09	0.0188^a^
A liquid–solid ratio	8.36	1	8.36	10.83	0.0072^b^
B‐extraction time	1.30	1	1.30	1.69	0.2203
C‐extraction temperature	2.13	1	2.13	2.76	0.1248
Residual	8.49	11	0.77		
Lack of fit	8.49	9	0.94	219.28	0.0045^b^
Pure error	0.01	2	0.00		

a Significant (α = 0.05).

b Extremely significant (α = 0.01).

**Table 4:** Analysis of Variance (ANOVA) for the reduced linear model (backward: α < 0.05) selected from Box‐Behnken designs

Source	Sum of Squares	*df*	Mean Square	F Value	p‐value
Model	8.36	1	8.36	9.11	0.0099^a^
A liquid–solid ratio	8.36	1	8.36	9.11	0.0099^a^
Residual	11.93	13	0.92		
Lack of fit	11.92	11	1.08	252.06	0.0040^a^
Pure error	0.01	2	0.00		

Significant (α = 0.05).

a Extremely significant (α = 0.01).

Tea‐leaf saponins yield was 9.61% ± 0.0034% under the extraction condition of 40 ml/g, 1 hr, and 80°C, only having a 0.52% relative standard deviation (R.S.D) with the value predicted by Box‐Behnken designs, which proved the predictive ability of the reduced linear model.

Water extraction was not a common method in extracting tea‐seed saponins from *C. oleifera* seed meals, although analysis of range's results (Chen et al., 2017) showed that the three influencing variables on tea‐seed saponins yields were in the following sequence: solid–liquid ratio > extraction temperature > extraction time, being consistent with the results of this study. Nonetheless, results of Lin‐jian Li, Wu, et al. (2016) were not the same: the sequence of these three variables was extraction temperature > extraction time > liquid–solid ratio. Three close liquid–solid ratio points set for the experiment with values of 5:1, 6:1 and 7:1 ml/g might be the cause of the inconsistency. Different extraction objects were also one of the reasons for the different influencing sequence of optimized variables.

### 3.2 Single‐variable experiments for liquid*–*solid ratio

A broader range of liquid–solid ratio was designed for the additional experiments with 1 hr and 80°C selected as extraction time and extraction temperature, considering the experimental operability and energy consumption.

**Figure 2:**
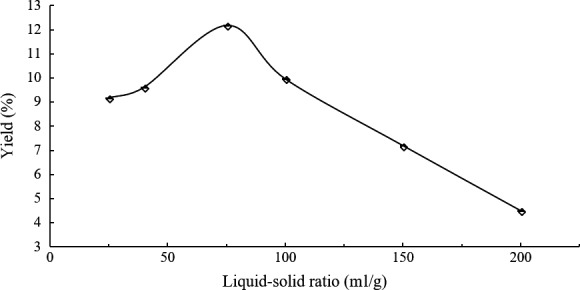
Effects of liquid–solid ratio on tea‐leaf saponins yields by single‐variable experiments

Figure 2 revealed the effect of liquid–solid ratio on tea‐leaf saponins yields by water extraction from 25 to 200 ml/g. Obviously, the tendency of liquid–solid ratio had an ascent at first followed with a decline afterward and the curve came to the peak at 75 ml/g. The optimal liquid–solid ratio of 75 ml/g was not similar as that in tea‐seed saponins extraction from *C. oleifera* seed meals, suggesting tea leaves required more water to obtain a higher yield of saponins than tea seeds, while too much water reduced the yield because the content of tea‐leaf saponins was a constant and the excessive water diluted the concentration of tea‐leaf saponins.

The tea‐leaf saponins yield of tea tree variety Longjing 43 reached 12.19% with liquid–solid ratio of 75 ml/g, extraction time of 1 hr, and extraction temperature of 80°C, being closer to the yield gotten with liquid–solid ratio of 40 ml/g, extraction time of 5 hr, and extraction temperature of 60°C in Box‐Behnken designs (Run 8, yield 12.18%), demonstrating an optimized liquid–solid ratio with a little higher extraction temperature could decrease the experiment time of water extraction and at the same time reduce the difficulty of experimental operation and the consumption of energy.

The 12.19% yield of tea‐leaf saponins was higher than the yield of tea‐seed saponins with the same method, water extraction based on optimizations, whose values were around 8.5% (Chen et al., 2017; Li, Wu, et al., 2016; Li, Yu, et al., 2016), which presented that it was feasible to extract saponins from aged tea leaves, in favor of the utilization of excessive tea leaves.

### 3.3 Influential factors on tea‐leaf saponins yields and contents

Under the same extraction conditions, tea‐leaf saponins yields of different tea tree varieties could be approximately equivalent to tea‐leaf saponins contents of different tea tree varieties; hence, as Table 5 presents, significant differences of tea‐leaf saponins contents existed between various tea tree varieties.

**Table 5:** Tea‐leaf saponins yield (%) of different tea tree varieties

Type	Longjing 43	Zhenong 117	Anji white tea	Zisun	Huangjinya	Jiukengzao
Yield (%)	12.19 ± 0.00^a^	15.30 ± 0.00^b^	15.80 ± 0.01^c^	16.14 ± 0.01^d^	20.18 ± 0.02^e^	22.65 ± 0.01^f^

Note

Different letters indicated significant differences (α = 0.05).

Table 6 provides the correlation coefficients between tea‐leaf saponins yields and three potential influential factors of tea trees both from Pearson's and Spearman's methods. These three factors are tea trees’ inherent properties, changing as tea tree varieties changed. Leaf type reflects the size of tea leaves, germination stage is the time tea trees grow new shoots and adaptability refers to the tea type, such as green tea, black tea, and oolong tea, that leaves picked from this tea tree variety are more suitable to produce.

**Table 6:** Correlations between tea‐leaf saponins yields and leaf type, germination stage along with adaptability of tea trees

	Leaf type	Germination stage	Adaptability
Pearson	0.7329	0.3819	−0.2735
Spearman	0.6547	0.4629	−0.2070

Because of the tea type Zhejiang Province mainly produced is green tea, adaptability of tea trees in this study lacked extensiveness and differences, might causing the inaccuracy of the correlation coefficient between tea‐leaf saponins yields and adaptability, nonetheless, it could be concluded that influential factors, leaf type, and germination stage had positive correlations with tea‐leaf saponin's contents and leaf type affected stronger, which indicated the tea tree with larger leaves as well as later germination stage enjoyed a higher content of tea‐leaf saponins and a higher yield under the same extraction conditions.

Saponin is a secondary metabolite of tea trees, and it is possible that its content increases as tea tree's leaf size increases and germination stage postpones. Nevertheless, the exact correlation coefficient still requires more experiments with a wider scale of tea tree varieties to analyze and come to a more accurate conclusion. All the same, it was feasible to extracting saponins from tea leaves, which benefitted the comprehensive utilization of tea leaves and contributed to solve the relative surplus of tea‐leaf production.

## 4 CONCLUSION

This study investigated and confirmed the feasibility of extracting saponins from tea (*Camellia sinensis*) leaves and optimized three major variables in water extraction, which were liquid–solid ratio, extraction time, and extraction temperature. After optimization by Box‐Behnken designs, the significant variable liquid–solid ratio was conducting an additional experiment to acquire a more accurate optimization using single‐variable method. Seventy‐five ml/g, 1 hr, and 80°C were optimal values and tea‐leaf saponins yield of tea tree variety Longjing 43 reached 12.19% ± 0.0030% after optimizations, higher than the yield of tea‐seed saponins from *Camellia oleifera* seed meals with the same extraction method (water extraction based on optimizations).

Factors affecting the yield of tea‐leaf saponins between different tea tree varieties were also analyzed and discussed. Correlation analyses between tea‐leaf saponins yields and three inherent properties of tea trees, leaf type, germination stage along with adaptability were carried out, and results showed that the variety of tea tree with larger leaves as well as later germination stage would have a higher content of tea‐leaf saponins as well as a higher yield under the same extraction conditions.

Confirming the feasibility of extracting saponins from tea leaves not only benefits to the in‐depth understanding of tea leaves, but also contributes to the comprehensive utilization of tea leaves, especially excessive aged tea leaves in autumn and winter, which was helpful to alleviate the relative surplus of tea‐leaf production in China.

## CONFLICT OF INTEREST

The authors declare that they do not have any conflict of interest.

## ETHICAL STATEMENT

This study does not involve any human or animal testing.

## References

[fsn3724-bib-0001] Cay, S. (2016). Enhancement of cadmium uptake by *Amaranthus caudatus*, an ornamental plant, using tea saponin. Environmental Monitoring and Assessment, 188(320), 1–8. 10.1007/s10661-016-5334-z 27142816

[fsn3724-bib-0002] Chen, X. , Xiao, D. , Huo, G. , Long, H. , & Liu, J. (2017). Extraction and purification of tea saponin and preparation of single angeloyl theasapogenol. Journal of the Chinese Cereals and Oils Association, 32(3), 117.

[fsn3724-bib-0003] Feng, J. , Chen, Y. , Liu, X. , & Liu, S. (2015). Efficient improvement of surface activity of tea saponin through Gemini‐like modification by straightforward esterification. Food Chemistry, 171, 272–279. 10.1016/j.foodchem.2014.08.125 25308669

[fsn3724-bib-0004] Gong, W. , Huang, Y. , Ji, A. , Peng, W. , Liu, C. , Zeng, Y. , … Sheng, J. (2017). Optimization of saponin extraction conditions with *Camellia sinensis* var. *assamica* seed and its application for natural detergent. Journal of the Science of Food and Agriculture, 98, 2312–2319. 10.1002/jsfa.8721 28990656

[fsn3724-bib-0005] Gong, Z. , Liang, D. , Zhang, Z. , & Xiao, W.‐J. (2013). Study on tea seed saponin extraction by microwave‐assisted method. Journal of Tea Science, 33(4), 358–363.

[fsn3724-bib-0006] He, J. , Wu, Z. , Zhang, S. , Zhou, Y. , Zhao, F. , Peng, Z. , & Hu, Z. (2014). Optimization of microwave‐assisted extraction of tea saponin and its application on cleaning of historic silks. Journal of Surfactants and Detergents, 17(5), 919–928. 10.1007/s11743-013-1523-8

[fsn3724-bib-0007] He, Z. , Zhang, H. , & Zhang, X. (2015). Research on extracting technology of tea saponin by ultrasonic assisting alcohol‐ammonia water. Natural Science Journal of Xiangtan University, 37(2), 80–85. https://doi.org/10.13715/j.cnki.nsjxu.2015.02.013

[fsn3724-bib-0008] Hu, J. , Nie, S. , Gong, Y. , Li, C. , & Xie, M. (2009). Application of response surface methodology for optimizing extraction technology of tea saponin. Food Science, 30(18), 106–109.

[fsn3724-bib-0009] Hu, J.‐L. , Nie, S.‐P. , Huang, D.‐F. , Li, C. , & Xie, M.‐Y. (2012). Extraction of saponin from *Camellia oleifera* cake and evaluation of its antioxidant activity. International Journal of Food Science and Technology, 47(8), 1676–1687. 10.1111/j.1365-2621.2012.03020.x

[fsn3724-bib-0010] Li, Y. , Wu, X. , Yang, L. , & Yi, Z. (2016). Study on the extraction of *Camellia* saponin and its application in the soap. Cereals and Oils, 29(10), 50–53.

[fsn3724-bib-0011] Li, L. , Yu, W. , Liu, G. , Liu, H. , Kuai, L. , Li, L. , & Chen, X. (2016). The extraction of tea saponin and its application in shampoos. Applied Chemical Industry, 45(1), 78–81.

[fsn3724-bib-0012] Liu, X. , Cao, L. , Wang, Q. , Zhang, X. , & Hu, X. (2017). Effect of tea saponin on phytoremediation of Cd and pyrene in contaminated soils by *Lolium multiflorum* . Environmental Science and Pollution Research, 24(23), 18946–18952. 10.1007/s11356-017-9515-2 28656573

[fsn3724-bib-0013] Liu, B. , Quan, C. , Huang, X. , Chen, Y. , & Zhang, Y. (2013). Optimization of ethanol extraction of tea saponin using response surface methodology. China Oils and Fats, 38(6), 84–86.

[fsn3724-bib-0014] Lu, W. , Ning, J. , Fang, S. , Jiang, S. , & Wei, H. (2012). Study on comprehensive extraction of tea polyphenol and tea saponin from tea flowers. Science and Technology of Food Industry, 11, 296–300. https://doi.org/10.13386/j.issn1002-0306.2012.11.081

[fsn3724-bib-0500] Luo, Y. (2013). Tea Tree Cultivation (4th ed.). Beijing: China Agriculture Press.

[fsn3724-bib-0015] Lv, X. , & Li, Z. (2005). Study on supercritical‐CO_2_ extraction of saponin from *Camellia oleifera* . Food and Fermentation Industries, 31(1), 23–26. https://doi.org/10.13995/j.cnki.11-1802/ts.2005.01.007

[fsn3724-bib-0016] Lv, X. , Qu, S. , Sun, X. , & Li, Z. (2005). Preliminary study on the capability of antioxidantion and scavenging free radicals of sasanquasaponins. Food Science, 26(11), 86–90.

[fsn3724-bib-0017] Makkar, H. P. S. , & Becker, K. (2007). Methods in Molecular Biology. Towota: Humana Press.

[fsn3724-bib-0018] Mao, L. , Qi, Y. , Sun, Z. , Zeng, Q. , & Yan, F. (2016). Extraction of tea saponin and surface performance of its solutions. Journal of Tianjin Polytechnic University, 35(5), 32–36. 10.3969/j.issn.1671-024x.2016.05.006

[fsn3724-bib-0019] Morikawa, T. , Matsuda, H. , Li, N. , Li, X. , & Yoshikawa, M. (2007). Bioactive saponins and glycosides. Part 29 Acylated oleanane‐type triterpene saponins: Theasaponins A6, A7, and B5 from the seeds of *Camellia sinensis* . Helvetica Chimica Acta, 90(12), 2342–2348. 10.1002/hlca.200790240

[fsn3724-bib-0020] Morikawa, T. , Nakamura, S. , Kato, Y. , Muraoka, O. , Matsuda, H. , & Yoshikawa, M. (2007). Bioactive saponins and glycosides. XXVIII. New triterpene saponins, foliatheasaponins I, II, III, IV, and V, from Tencha (the leaves of *Camellia sinensis*). Chemical and Pharmaceutical Bulletin (Tokyo), 55(2), 293–298. 10.1248/cpb.55.293 17268104

[fsn3724-bib-0021] Peng, Y. , Zhou, J. , & Guo, H. (2009). Study oil extraction of tea saponin by microwave‐assisted method. Cereals and Oils, 3, 27–29.

[fsn3724-bib-0022] Qi, X. , & Zhang, S. (2014). Study on ultrasonic assisted water extraction of tea saponin and application of its solution. Journal of Food Science and Technology, 32(1), 59–64. 10.3969/j.issn.2095-6002.2014.01.011

[fsn3724-bib-0023] Ribeiro, B. D. , Coelho, M. A. Z. , Rebelo, L. P. N. , & Marrucho, I. M. (2013). Ionic liquids as additives for extraction of saponins and polyphenols from mate (*Ilex paraguariensis*) and tea (*Camellia sinensis*). Industrial and Engineering Chemistry Research, 52(34), 12146–12153. 10.1021/ie400529h

[fsn3724-bib-0024] Sagesaka, Y. M. , Uemura, T. , Watanabe, N. , Sakata, K. , & Uzawa, J. (1994). A new glucuronide saponin from tea leaves (*Camellia sinensis* var. *sinensis*). Bioscience, Biotechnology and Biochemistry, 58(11), 2036–2040. 10.1080/bbb.58.2036 7765596

[fsn3724-bib-0025] Shi, G. , Wang, H. , Hua, R. , Zhou, B. , Xu, J. , & Deng, L. (2011). Technical study on ethanol‐ammonia water extraction of tea saponin from *Camellia* cakes. Modern Chemical Industry, 31(4), 37–40.

[fsn3724-bib-0026] Sun, J. , Cai, C. , Liang, R. , & Yang, C. (2017). Purification of tea saponin and its inhibition on tyrosinase activity. China Surfactant Detergent and Cometics, 47(6), 312–316, 340. https://doi.org/10.13218/j.cnki.csdc.2017.06.003

[fsn3724-bib-0027] Wan, X. (2011). Tea Biochemistry, (3rd ed.). Beijing: China Agriculture Press.

[fsn3724-bib-0028] Wang, X. (2013). Process optimization research on aqueous enzymatic extraction of tea‐seed oil with response surface analysis. Journal of the Chinese Cereals and Oils Association, 28(5), 40–43, 48.

[fsn3724-bib-0029] Wang, W. , Chen, C. , Zhang, B. , Huang, J. , Wu, X. , Xu, Y. , … Wu, Q. (2014). Method of abstracting tea‐saponin with secondary fermentation. Chinese Agricultural Science Bulletin, 30(30), 296–301.

[fsn3724-bib-0030] Wang, Q. , Liu, X. , Zhang, X. , Hou, Y. , Hu, X. , Liang, X. , & Chen, X. (2016). Influence of tea saponin on enhancing accessibility of pyrene and cadmium phytoremediated with *Lolium multiflorum* in co‐contaminated soils. Environmental Science and Pollution Research, 23(6), 5705–5711. 10.1007/s11356-015-5784-9 26581690

[fsn3724-bib-0031] Wen, L. , Lu, W. , Jiang, Q. , Yan, L. , & Fang, D. (2011). Toxicity irritation and bacteriostasis of tea saponin. China Oils and Fats, 36(6), 58–60.

[fsn3724-bib-0032] Wu, X. , Huang, R. , Huang, C. , Wang, B. , & Song, H. (2017). Study on properties of tea saponin, a kind of natural non‐ionic surfactant. China Cleaning Industry, 2, 38–42.

[fsn3724-bib-0033] Xiong, W. , Li, X. , Fu, J. , Wang, H. , Zhu, R. , & Han, X. (2016). Synchronous extraction of procyanidins and saponin from episperm of tea seeds. Food Research and Development, 37(20), 28–32. 10.3969/j.issn.1005-6521.2016.20.008

[fsn3724-bib-0034] Yan, X. , Wei, J. , Xu, J. , Li, J. , & Guo, X. (2014). Study on antibacterial activities of tea polyphenols and tea saponin and their blends. Science and Technology of Food Industry, 35(22), 159–162. https://doi.org/10.13386/j.issn.1002-0306.2014.22.026

[fsn3724-bib-0035] Yang, P. , Zhou, M. , Zhou, C. , Wang, Q. , Zhang, F. , & Chen, J. (2015). Separation and purification of both tea seed polysaccharide and saponin from *Camellia* cake extract using macroporous resin. Journal of Separation Science, 38(4), 656–662. 10.1002/jssc.201401123 25491912

[fsn3724-bib-0036] Ye, M. , Sun, M. , Wan, J. , Fang, G. , Li, H. , Hu, F. , … Kengara, F. O. (2015). Evaluation of enhanced soil washing process with tea saponin in a peanut oil‐water solvent system for the extraction of PBDEs/PCBs/PAHs and heavy metals from an electronic waste site followed by vetiver grass phytoremediation. Journal of Chemical Technology and Biotechnology, 90(11), 2027–2035. 10.1002/jctb.4512

[fsn3724-bib-0037] Ye, M. , Sun, M. , Xie, S. , Liu, K. , Feng, Y. , Zhao, Y. , … Jiang, X. (2017). Feasibility of tea saponin‐enhanced soil washing in a soybean oil‐water solvent system to extract PAHs/Cd/Ni efficiently from a coking plant site. Pedosphere, 27(3), 452–464. 10.1016/S1002-0160(17)60341-2

[fsn3724-bib-0038] Yi, X. , Liu, R. , Xiao, Z. , Wu, H. , Gu, Z. , Li, J. , & Li, C. (2016). Research on tea oil and tea saponin extracted by *n*‐butyl alcohol from pressed *Camellia* cake. Journal of the Chinese Cereals and Oils Association, 31(4), 67–71.

[fsn3724-bib-0039] Yoshikawa, M. , Morikawa, T. , Li, N. , Nagatomo, A. , Li, X. , & Matsuda, H. (2005). Bioactive saponins and glycosides. XXIII. Triterpene saponins with gastroprotective effect from the seeds of *Camellia sinensis*–theasaponins E3, E4, E5, E6, and E7. Chemical and Pharmaceutical Bulletin, 53(December), 1559–1564. 10.1248/cpb.53.1559 16327189

[fsn3724-bib-0040] Yoshikawa, M. , Morikawa, T. , Nakamura, S. , Li, N. , Li, X. , & Matsuda, H. (2007). Bioactive saponins and glycosides. XXV. Acylated oleanane‐type triterpene saponins from the seeds of tea plant (*Camellia sinensis*). Chemical and Pharmaceutical Bulletin (Tokyo), 55(1), 57–63. 10.1248/cpb.55.57 17202702

[fsn3724-bib-0041] Yu, H. , Chen, H. , Wu, B. , & Ren, W. (2013). Optimization of extraction process for tea saponin from *Camellia oleifera* seed meal with *n*‐propanol. Food Science, 34(2), 58–62.

[fsn3724-bib-0042] Zhan, Y. , & Xie, Y. (2012). Study on the extraction technology of tea saponin from *Camellia chekiang‐oleosa* Hu tea seed cake. Journal of Anhui Agricultural Science, 40(1), 104–105. https://doi.org/10.13989/j.cnki.0517-6611.2012.01.055

[fsn3724-bib-0043] Zhang, L. , Hu, J. , Li, J. , & Deng, Z. (2012). Extraction of tea saponin by ultrasonic‐assisted method from the tea seed residue after tea oil extraction. Journal of Anhui Agricultural Science, 40(18), 9888–9891.

[fsn3724-bib-0044] Zhang, K. , Qian, H. , Zhang, T. , & Fan, L. (2003). Study on extraction of tea saponin from out cake of *Camellia oleifera* seeds. Food Science and Technology, 10, 68–70.

[fsn3724-bib-0045] Zhang, Y. , & Wang, L. (2014). Extraction of tea saponin by enzymatic hydrolysis method. Journal of Henan University of Technology (Natural Science Edition), 35(5), 17–22.

[fsn3724-bib-0046] Zhao, S. , Xue, Z. , Yang, C. , & Wei, M. (2010). Optimization of extraction conditions of tea saponin and its antifungi effects. China Oils and Fats, 35(5), 64–67.

[fsn3724-bib-0047] Zhou, H. , & Yang, D. (2016). Enzymatic extraction technology and purification method of tea saponin. Jiangsu Agricultural Sciences, 44(5), 362–364. https://doi.org/10.15889/j.issn.1002-1302.2016.05.106

[fsn3724-bib-0048] Zhu, X. , Lin, H. , Chen, X. , Xie, J. , & Wang, P. (2011). Optimization of homogenate extraction of theasaponin from *Camellia oleifera* Abel. meal. Transactions of the CSAE, 27(Suppl. 1), 402–406. 10.3969/j.issn.1002-6819.2011.z1.076

